# Independent effect of body mass index variation on amyloid-β positivity

**DOI:** 10.3389/fnagi.2022.924550

**Published:** 2022-07-22

**Authors:** Sung Hoon Kang, Jong Hyuk Kim, Yoosoo Chang, Bo Kyoung Cheon, Yeong Sim Choe, Hyemin Jang, Hee Jin Kim, Seong-Beom Koh, Duk L. Na, Kyunga Kim, Sang Won Seo

**Affiliations:** ^1^Department of Neurology, Samsung Medical Center, School of Medicine, Sungkyunkwan University, Seoul, South Korea; ^2^Neuroscience Center, Samsung Medical Center, Seoul, South Korea; ^3^Department of Neurology, Korea University Guro Hospital, Korea University College of Medicine, Seoul, South Korea; ^4^Department of Digital Health, SAIHST, Sungkyunkwan University, Seoul, South Korea; ^5^Center for Cohort Studies, Total Healthcare Center, Kangbuk Samsung Hospital, School of Medicine, Sungkyunkwan University, Seoul, South Korea; ^6^Department of Health Sciences & Technology, SAIHST, Sungkyunkwan University, Seoul, South Korea; ^7^Biomedical Statistics Center, Research Institute for Future Medicine, Samsung Medical Center, Seoul, South Korea; ^8^Department of Data Convergence and Future Medicine, School of Medicine, Sungkyunkwan University, Seoul, South Korea; ^9^Samsung Alzheimer Research Center, Center for Clinical Epidemiology Medical Center, Seoul, South Korea; ^10^Department of Intelligent Precision Healthcare Convergence, SAIHST, Sungkyunkwan University, Seoul, South Korea

**Keywords:** amyloid-**β** (A**β**), body mass index (BMI), BMI change, BMI variability, Alzheimer’s disease

## Abstract

**Objectives:**

The relationship of body mass index (BMI) changes and variability with amyloid-β (Aβ) deposition remained unclear, although there were growing evidence that BMI is associated with the risk of developing cognitive impairment or AD dementia. To determine whether BMI changes and BMI variability affected Aβ positivity, we investigated the association of BMI changes and BMI variability with Aβ positivity, as assessed by PET in a non-demented population.

**Methods:**

We retrospectively recruited 1,035 non-demented participants ≥50 years of age who underwent Aβ PET and had at least three BMI measurements in the memory clinic at Samsung Medical Center. To investigate the association between BMI change and variability with Aβ deposition, we performed multivariable logistic regression. Further distinctive underlying features of BMI subgroups were examined by employing a cluster analysis model.

**Results:**

Decreased (odds ratio [OR] = 1.68, 95% confidence interval [CI] 1.16–2.42) or increased BMI (OR = 1.60, 95% CI 1.11–2.32) was associated with a greater risk of Aβ positivity after controlling for age, sex, APOE e4 genotype, years of education, hypertension, diabetes, baseline BMI, and BMI variability. A greater BMI variability (OR = 1.73, 95% CI 1.07–2.80) was associated with a greater risk of Aβ positivity after controlling for age, sex, APOE e4 genotype, years of education, hypertension, diabetes, baseline BMI, and BMI change. We also identified BMI subgroups showing a greater risk of Aβ positivity.

**Conclusion:**

Our findings suggest that participants with BMI change, especially those with greater BMI variability, are more vulnerable to Aβ deposition regardless of baseline BMI. Furthermore, our results may contribute to the design of strategies to prevent Aβ deposition with respect to weight control.

## Introduction

A large amount of evidence suggests that body mass index (BMI) is associated with the risk of developing cognitive impairment. Specifically, mid-life obesity increases the risk of cognitive impairment (Kivipelto et al., 2005; Fitzpatrick et al., 2009; Tolppanen et al., 2014). Previous studies have also shown that being underweight in later life may be associated with an increased risk of dementia (Tolppanen et al., 2014; Bell et al., 2017), and the acceleration of cortical atrophy (Kim H. et al., 2015; Kim et al., 2019). Furthermore, larger BMI changes were also associated with a higher risk of conversion to dementia in patients with mild cognitive impairment (MCI), (Ye et al., 2016) and these changes had deleterious effects on the cognitive function in the non-demented elderly (Giudici et al., 2019).

Amyloid-β (Aβ) deposition in the brain is an important pathological hallmark of Alzheimer’s disease (AD), which is the most common cause of dementia. According to the Aβ cascade hypothesis, Aβ deposition in the brain occurs at a very early stage. In fact, Aβ deposition in the brain precedes the development of AD dementia by 10–20 years. Furthermore, non-demented participants with Aβ deposition were more converted to dementia than those without Aβ deposition (Villemagne et al., 2011; Rowe et al., 2013; Ye et al., 2018). Therefore, Aβ positivity is a crucial predictor of AD prognosis in non-demented individuals.

Recently, being underweight or obese in mid-life was found to be associated with an increased risk of Aβ deposition (Gottesman et al., 2017; Lee et al., 2020; Möllers et al., 2021), and individuals who are underweight in later life were also at an increased risk of Aβ deposition (Vidoni et al., 2011; Ewers et al., 2012; Hsu et al., 2016; Thirunavu et al., 2019). However, since previous studies investigated the association of BMI with Aβ deposition at a single time point, these studies could not evaluate the effects of BMI variability on Aβ deposition. Considering that BMI changes and variability is closely associated with a new onset of diabetes, cardiovascular disease, atrial fibrillation, and higher mortality Bangalore et al., 2017; Lim et al., 2019; Sponholtz et al., 2019), it is reasonable to expect that BMI changes and variability may be important risk factors for Aβ deposition. Therefore, by studying the association of BMI changes and variability with Aβ deposition, it could be identified whether BMI changes and variability is specifically related to AD, especially in the early stage of AD pathobiology. Furthermore, this study may provide the importance of rigorous strategies for weight maintenance to prevent Aβ deposition in non-demented individuals.

Thus, the goal of our study was to investigate the influence of BMI changes and BMI variability on Aβ positivity in a non-demented population. Furthermore, to determine the complex relationships among BMI changes, BMI variability, and Aβ positivity, we evaluated the distinguishable BMI subgroups classified by the cluster analysis model. We hypothesized that decreased BMI and greater BMI variability would have deleterious effects on Aβ deposition.

## Materials and methods

### Participants

Our study was designed as a retrospective cohort study. We recruited 1,546 non-demented participants ≥50 years of age [611 with normal cognition and 935 with mild cognitive impairment (MCI)] from the memory clinic in the Department of Neurology at Samsung Medical Center (SMC) in Seoul, Korea. The participants had undergone Aβ PET *between August 2015 and* August 2020. All participants underwent a comprehensive dementia work-up including the standardized cognitive assessment battery (Kang et al., 2019), APOE genotyping, and brain MRI. We excluded participants who had any of the following conditions: severe white matter hyperintensities based on the Fazekas scale; structural lesions such as brain tumor, large territorial infarct, and intracranial hemorrhage; other causes of neurodegenerative diseases including Lewy body dementia, Parkinson’s disease, cortico-basal syndrome, progressive supranuclear palsy, and frontotemporal dementia.

All participants with normal cognition fulfilled the following conditions: (1) no medical history that is likely to affect cognitive function based on Christensen’s criteria (Christensen et al., 1991); (2) no objective cognitive impairment from a comprehensive neuropsychological test battery on any cognitive domain (at least −1.0 SD above age-adjusted norms on any cognitive tests); and (3) independence in activities of daily living. All participants with MCI fulfilled Petersen’s criteria with the following modifications (Petersen, 2011; Jeong et al., 2020): (1) subjective cognitive complaints by the participants or caregiver; (2) objective memory impairment below −1.0 SD on verbal or visual memory tests; (3) no significant impairment in activities of daily living; (4) non-demented.

The institutional review board of the SMC approved this study. Written informed consent was obtained from all participants.

#### Amyloid-β PET acquisition

All participants underwent Aβ PET (^18^F-florbetaben [FBB] PET and ^18^F-flutemetamol [FMM] PET) scans at SMC using a Discovery STe PET/CT scanner (GE Medical Systems, Milwaukee, WI, United States). For FBB or FMM PET, a 20-min emission PET scan in dynamic mode (consisting of 4 min × 5 min frames) was performed 90 min after an injection of a mean dose of 311.5 MBq FBB and 197.7 MBq FMM, respectively. Three-dimensional PET images were reconstructed in a 128 × 128 × 48 matrix with a 2 mm × 2 mm × 3.27 mm voxel size using the ordered-subsets expectation maximization algorithm (FBB, iteration = 4 and subset = 20; FMM, iteration = 4 and subset = 20).

#### Amyloid-β PET assessment

Aβ positivity on PET scans was determined using visual reads in the primary analyses. Specifically, Aβ PET images were rated by two experienced doctors (one nuclear medicine physician and one neurologist) who were blinded to the clinical information, and the images were dichotomized as either Aβ-positive (Aβ+) or Aβ-negative (Aβ−) using visual reads. They discussed any discordant results regarding Aβ positivity to achieve consensus. The FBB PET scan was regarded as positive if the Aβ plaque load was visually rated as 2 or 3 on the brain amyloid plaque load scoring system, and the FMM PET scan was considered positive if one of five brain regions (frontal, parietal, posterior cingulate and precuneus, striatum, and lateral temporal lobes) systematically reviewed using FMM PET was positive in either hemisphere (Kang et al., 2021). Representative PET images in participants with Aβ+ and Aβ− were shown in Supplementary Figure 1. Inter-rater agreement of PET interpretation was excellent for Aβ positivity at the subject level (kappa score = 0.84). In addition, visual rating was highly concordant with a standardized uptake value ratio (SUVR) cutoff categorization for Aβ positivity (93.5% for FBB and 91.6% for FMM).

#### Amyloid-β PET quantification using Centiloid values

For the sensitivity analyses, Aβ positivity on PET scans was determined using Centiloid (CL) cutoff-based categorization. We used a CL method previously developed by our group (Cho et al., 2020) to standardize the quantification of Aβ PET images obtained using different ligands. The CL method for FBB and FMM PET enables the transformation of the SUVR of FBB and FMM PET to CL values directly without conversion to the ^11^C-labeled Pittsburgh compound SUVR.

There are three steps to obtain CL values (Cho et al., 2020): (1) preprocessing of PET images, (2) determination of CL global cortical target volume of interest (CTX VOI), and (3) conversion of SUVR to CL values. First, to preprocess the Aβ PET images, PET images were co-registered to each participant’s MR image and then normalized to a T1-weighted MNI-152 template using the SPM8 unified segmentation method (Klunk et al., 2015). We used T1-weighted MRI correction with the N3 algorithm only for intensity non-uniformities (Sled et al., 1998), without applying corrections to the PET images for brain atrophy or partial volume effects. Second, we used the FBB-FMM CTX VOI defined as areas of AD-specific brain Aβ deposition in our previous study (Cho et al., 2020). Briefly, to exclude areas of aging-related brain Aβ deposition, the FBB-FMM CTX VOI was generated by comparing SUVR parametric images (with the whole cerebellum as a reference area) between 20 typical patients with AD-related cognitive impairment (AD-CTX) and 16 healthy elderly participants (EH-CTX) who underwent both FBB and FMM PET scans. To generate the FBB-FMM CTX VOI, the average EH-CTX image was subtracted from the average AD-CTX image. We then defined the FBB-FMM CTX VOI as areas of AD-related brain Aβ accumulation common to both FBB and FMM PET. Finally, the SUVR values of the FBB-FMM CTX VOI were converted to CL units using the CL conversion equation. The CL equation was derived from the FBB-FMM CTX VOI separately for FBB and FMM PET and applied to FBB and FMM SUVR.

To determine the participants’ CL cutoff-based Aβ positivity, we applied the optimal cutoff value derived using a k-means cluster analysis in 527 independent samples of participants with normal cognition. The cutoff value was set at 27.08, representing the 95th percentile of the lower cluster (Villeneuve et al., 2015), and the whole cerebellum was used as a reference region (Kim et al., 2021).

#### Body mass index acquisition

For each participant, BMI data were obtained by backtracking their weight and height records in clinical data warehouse of SMC, which were measured at all visits within 3 years after inspecting Aβ PET until March 2000 (Supplementary Figure 2). We excluded 511 participants who did not have at least three BMI measurements. A total of 1,035 non-demented participants (409 with normal cognition and 626 with MCI) were included in the present study.

#### Body mass index assessment

Baseline BMIs was defined as the farthest past measurement from the Aβ PET scan (Supplementary Figure 3). Participants were then categorized into three subgroups according to their baseline BMI values based on the World Health Organization’s recommendations for Asian populations: underweight (BMI < 18.5 kg/m^2^), normal weight (18.5–24.9 kg/m^2^), obesity (BMI ≥ 25 kg/m^2^) (WHO Expert Consultation, 2004).

Follow-up BMI was defined as the closest measurement to the Aβ PET scan (Supplementary Figure 3). We calculated BMI change as the change rate by taking the difference between baseline and follow-up BMIs and dividing it by duration. Using the 1st and 3rd quantiles (Q1 and Q3) of BMI changes, we characterized participants as increased (≥Q3), stable, and decreased (≤Q1).


$$B\,M\,I\,c\,h\,a\,n\,g\,e=\frac{B\,M\,I_{f}-B\,M\,I_{b}}{D\,u\,r\,a\,t\,i\,o\,n}$$

*B_b_: baseline BMI*, *B_f_: follow-up BMI*, *Duration: period between B*_*b*_ *and* *B*_*f*_

BMI values at three or more time points were used to obtain BMI variability (Supplementary Figure 3). Six measures of variability were considered: standard deviation (SD), coefficient of variation (CV), variability independent of the mean (VIM), residual standard deviation (RSD), average real variability (ARV), and successive variability (SV). These variability measures have different characteristics, and their formulae are shown in Supplementary Table 1. While SD was known to suffer from the bias due to the correlation with the mean value, CV and VIM were developed to avoid this bias. RSD was designed to take the time effect into account when assessing variability among longitudinal measurements. ARV and SV were calculated based on the differences between the consecutive measurements. BMI variability values were obtained based on each of six variability measures. While we used VIM-based variability for the primary association analysis, we considered all six measures to identify underlying patterns in longitudinal BMI values *via* the cluster analysis. In the association analysis, participants were classified into subgroups with non-high variability versus with high variability according to the upper 15-percentile of BMI variability values as a threshold.

#### Statistical analyses

We used independent *t*-tests and chi-square tests to compare the demographic and clinical characteristics of Aβ+ and Aβ− groups. To investigate the association between BMI change and variability with Aβ deposition, we performed multivariable logistic regression using BMI change and variability as predictors after controlling for age, sex, APOE e4 genotype, years of education, hypertension, diabetes, and baseline BMI. To further validate the association between BMI change and variability with Aβ deposition, we performed multivariable logistic regression using CL cutoff-based Aβ positivity as an outcome rather than visually rated Aβ positivity.

Further distinctive underlying patterns in longitudinal BMI values were recognized by employing clustering algorithms including Gaussian mixture model (GMM), k-means clustering, and self-organizing map (Bilmes, 1998; Yang et al., 2012). First, we examined the correlations among BMI features, such as baseline BMI, BMI change and six BMI variability measures, to select features that would play complementary roles in clustering (Supplementary Figure 4). Second, the similarity between participants was assessed *via* the Euclidean distance after each selected feature was scaled through standardization. Finally, we used GMM to identify clusters, each of which consisted of participants with similar BMI patterns, because it can be used when more fine-grained workload characterization and analysis are required (Patel and Kushwaha, 2020). The optimal number of clusters was determined by validating clustering results with clinical interpretability as well as the silhouette index, Akaike information criterion (AIC) and Bayesian information criterion (BIC) measures. The identified clusters were further validated by examining the consistency with clustering results from the other algorithms. To investigate the association between the identified clusters (called as BMI subgroups) and Aβ deposition, we used multivariable logistic regression after controlling for age, sex, APOE e4 genotype, years of education, hypertension, and diabetes.

All reported *p*-values were two-sided, and the significance level was set at 0.05. All analyses were performed using SPSS version 25.0 and R version 4.3.0 (Institute for Statistics and Mathematics, Vienna, Austria^1^).

### Data availability

Anonymized data for our analyses presented in the present report are available upon request from the corresponding authors.

## Results

### Clinical characteristics of participants

Among the 1,035 participants, 579 individuals were Aβ− and 456 were Aβ+ (Table 1). Participants who were Aβ+ were more likely to be older (age, 70.6 ± 7.4 years) than those who were Aβ− (age, 68.5 ± 8.6 years, *p* < 0.001). Participants who were Aβ+ had a higher frequency of APOE e4 genotype (62.7% vs. 48.5%, *p* < 0.001) but a lower frequency of hypertension (39.0% vs. 48.5%, *p* = 0.003) and diabetes (13.2% vs. 24.4%, *p* < 0.001) than those who were Aβ−.

**TABLE 1:** Demographic and clinical characteristics of study participants.

	Aβ (−) (*n* = 579)	Aβ (+) (*n* = 456)	*P*-value
**Demographics**			
Gender, females	306 (52.8%)	261 (57.2%)	0.179
Age, years	68.5 ± 8.6	70.5 ± 7.4	0.001
Education, years	12.1 ± 4.8	12.0 ± 4.5	0.843
**Clinical characteristics**			
APOE e4 carrier	127 (21.9%)	286 (62.7%)	<0.001
Hypertension	281 (48.5%)	178 (39.0%)	0.003
Diabetes	141 (24.4%)	60 (13.2%)	<0.001
Follow-up years	6.1 ± 4.8	4.8 ± 4.2	<0.001
Baseline BMI			<0.001
Normal	334 (57.7%)	317 (69.5%)	
Obese	238 (41.1%)	121 (26.5%)	
Underweight	7 (1.2%)	18 (3.9%)	

Aβ, amyloid-β; BMI, body mass index. Values are presented as mean ± SD or *n* (%).

### Association of amyloid-β positivity with body mass index change and variability

Decreased BMI (odds ratio [OR] = 1.68, 95% confidence interval [CI] 1.16–2.42) was associated with a greater risk of Aβ positivity after controlling for age, sex, APOE e4 genotype, years of education, hypertension, diabetes, baseline BMI, and BMI variability (Table 2). Increased BMI (OR = 1.60, 95% CI 1.11–2.32) was also associated with a greater risk of Aβ positivity (Table 2).

**TABLE 2:** Association of Aβ positivity with BMI change and variability.

	OR (95% CI)*	*p*
**Visually rated Aβ positivity**		
**BMI change**		
Stable	1 (ref.)	
Decreased	1.68 (1.16–2.42)	0.006
Increased	1.60 (1.11–2.32)	0.012
**BMI variability**		
Normal	1 (ref.)	
High	1.73 (1.07–2.80)	0.025
**CL cutoff-based Aβ positivity**		
**BMI change**		
Stable	1 (ref.)	
Decreased	1.56 (1.06–2.29)	0.025
Increased	1.58 (1.06–2.34)	0.024
**BMI variability**		
Normal	1 (ref.)	
High	2.12 (1.28–3.51)	0.003

Aβ, amyloid-β; BMI, body mass index; OR, odds ratio; CI, confidence; CL, Centiloid. *Adjusted ORs for Aβ positivity were obtained by logistic regression analysis with BMI change and BMI variability together as predictors after controlling for age, sex, APOE e4 genotype, years of education, hypertension, diabetes, and baseline BMI.

A greater BMI variability (OR = 1.73, 95% CI 1.07–2.80) was associated with a greater risk of Aβ positivity after controlling for age, sex, APOE e4 genotype, years of education, hypertension, diabetes, baseline BMI, and BMI change (Table 2).

### Body mass index subgroups using Gaussian mixture model cluster analysis

**FIGURE 1:**
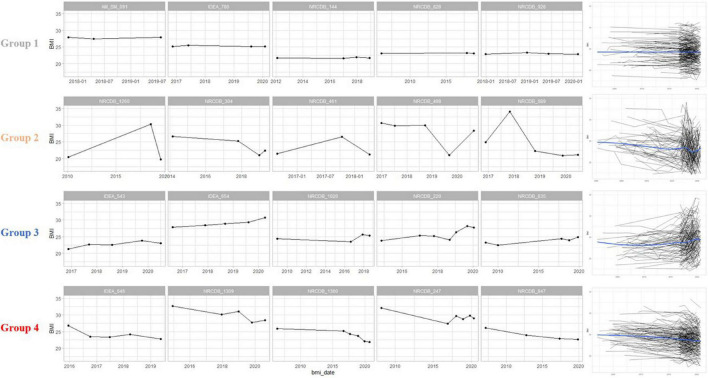
Body mass index (BMI) patterns of representative participants in each group. Group 1 had a constant pattern of both BMI change and variability. Group 2 showed a constant BMI change and some variability. Group 3 was a cluster with increasing BMI changes and high variability. Group 4 was a cluster with a decreasing BMI changes and high variability. The graphs on the right are spaghetti plots representing the BMI pattern of all patients in each group, and a blue line representing the trend of the group. BMI, body mass index.

Based on BMI baseline, change, and variability measures, four subgroups were created by GMM and validated with the Silhouette index, AIC and BIC (Supplementary Figure 5). The BMI characteristics for each subgroup were summarized in Supplementary Table 2. While the BMI baselines were very similar across all subgroups, BMI change and variability measures were different between subgroups. Because the mean change and variability measures were relatively small in subgroup 1 compared to other subgroups, we characterized subgroup 1 as the stable subgroup. Likewise, subgroups 2, 3, and 4 were characterized, respectively, as the subgroup having stable change with some variability; the subgroup having slightly increased BMI with some variability; the subgroup having decreased BMI with more variability. Longitudinal BMI patterns of representative patients in each subgroup are shown in Figure 1.

Compared to the stable subgroup 1, the subgroups showing greater BMI variability had a greater risk of Aβ positivity, especially when BMI was decreasing: subgroup 2 (OR = 1.49, 95% CI 1.05–2.13), subgroup 3 (OR = 1.49, 95% CI 1.01–2.20), and subgroup 4 (OR = 2.39, 95% CI 1.37–4.16) (Table 3).

**TABLE 3:** Association of Aβ positivity with BMI subgroup.

BMI subgroup*	OR (95% CI)*	*p*
1	1 (ref.)	
2	1.49 (1.05–2.13)	0.027
3	1.49 (1.01–2.20)	0.044
4	2.39 (1.37–4.16)	0.002

Aβ, amyloid-β; BMI, body mass index; OR, odds ratio; CI, confidence interval. *Adjusted ORs for Aβ positivity were obtained by logistic regression analysis with BMI subgroup as a predictor after controlling for age, sex, APOE e4 genotype, years of education, hypertension, and diabetes.

### Association of Centiloid cutoff-based amyloid-β positivity with body mass index change and variability

For CL cutoff-based Aβ positivity, decreased BMI (OR = 1.56, 95% CI 1.06–2.29) was associated with a greater risk of Aβ positivity after controlling for age, sex, APOE e4 genotype, years of education, hypertension, diabetes, baseline BMI, and BMI variability (Table 2). Increased BMI (OR = 1.58, 95% CI 1.06–2.34) was also associated with a greater risk of Aβ positivity (Table 2). Higher BMI variability (OR = 2.12, 95% CI 1.28–3.51) was associated with a greater risk of Aβ positivity after controlling for age, sex, APOE e4 genotype, years of education, hypertension, diabetes, baseline BMI, and BMI change (Table 2).

## Discussion

We systematically investigated the association of Aβ positivity with BMI change and variability in a relatively large group of participants who did not have dementia. In the present study, we found that BMI change (increased or decreased) was associated with a greater risk of Aβ positivity, regardless of baseline BMI status. Furthermore, we noted that greater variability in BMI predicted a greater risk of Aβ positivity despite baseline BMI status and BMI change. Finally, our cluster analysis identified BMI subgroups with specific patterns of BMI change and variability, which eventually showed a greater risk of Aβ positivity. Taken together, our findings suggest that participants with BMI change, especially those with greater BMI variability, are more vulnerable to Aβ deposition regardless of baseline BMI. Furthermore, given the paucity of modifiable risk factors for the development of Aβ, our results may contribute to the design of strategies to prevent Aβ deposition with respect to weight control.

Our major finding was that BMI change (increased or decreased) was associated with a greater risk of Aβ positivity, regardless of baseline BMI status. Considering that Aβ deposition is associated with the development of dementia, our findings are supported by previous epidemiologic studies (Ye et al., 2016; Giudici et al., 2019). Specifically, a previous study suggested that patients with MCI who had larger BMI changes are more likely to convert to probable AD dementia regardless of baseline BMI status (Ye et al., 2016). In another study, cognitively unimpaired individuals with decreased BMI were also reported to be at an increased risk of cognitive decline over a 5-year follow-up (Giudici et al., 2019). Furthermore, in agreement with our finding, recent study identified that decreased BMI or unstable BMI was associated with Aβ positivity in non-demented individuals (Buchman et al., 2021; Lane et al., 2021). Altogether, there is no exact pathobiology to explain these associations. However, as the elderly age, there is a loss of muscle mass and gain in visceral fat (Al-Sofiani et al., 2019). Therefore, it is reasonable to expect that participants with decreased BMI and increased BMI might reflect decreased muscle mass and increased visceral fat, respectively. In fact, some studies have investigated the relationship between the progression of sarcopenia and Aβ deposition (Maltais et al., 2019). Aβ deposition was found to be associated with sarcopenia, which might be mediated by an increased systemic inflammatory reaction (Yaffe et al., 2004; Maltais et al., 2019). Alternatively, decreased BMI might be an early reflective symptom of Aβ pathology (Grundman et al., 1996). In fact, a recent study revealed that the Aβ burden was associated with a prospective BMI decline in individuals with normal cognition (Rabin et al., 2020). On the other hand, regarding increased BMI, increased visceral fat deposition might affect brain atrophy and Aβ deposition (Kim H. J. et al., 2015) through several potential mechanisms, including increased insulin resistance (Jack et al., 2013; Luchsinger et al., 2013), lower levels of adipose-derived hormones (Montague et al., 1998), and a larger pattern of proatherogenic gene expression (Yao et al., 2012). Further studies are needed to examine the independent effects of specific body composition on Aβ deposition.

Another major novel finding was that greater BMI variability was associated with a greater risk of Aβ positivity, regardless of baseline BMI status and BMI changes. For example, even if the BMI measured 5 years ago and the current BMI are at the same level, there is a higher possibility of Aβ deposition if there is a high variation in BMI within the 5 years. We reviewed the literature regarding between BMI and AD related outcome (Supplementary Table 3). To the best of our knowledge, the association between BMI variability and Aβ positivity has not been thoroughly investigated. These findings offer new insight into an important role of BMI change and variability in non-demented individuals, and evidence that unstable and higher variability in BMI may be early manifestation related to Aβ deposition before developing dementia. The exact pathobiology of why greater BMI variation is detrimental to Aβ deposition remains unclear. However, there has been growing evidence that greater BMI variation is closely associated with a new onset of diabetes, cardiovascular disease, atrial fibrillation, and higher mortality (Bangalore et al., 2017; Lim et al., 2019; Sponholtz et al., 2019). One possible mechanism of these findings might be gene alternation. Specifically, the anti-aging gene Sirtuin 1 repression is associated with the onset of diabetes, cardiovascular disease and sarcopenia (Martins, 2016, 2017, 2018), which in turn leads to BMI variation, eventually resulting in Aβ deposition. Another possible explanation is that these medical diseases might result in both greater BMI variation and Aβ positivity, although we excluded participants with severe medical diseases using Christensen’s criteria (Christensen et al., 1991). Alternatively, regarding the greater BMI variability without medical diseases, even though there is a repeated occurrence and recovery of sarcopenia and visceral obesity, the accumulation of the remaining detrimental effects might affect Aβ deposition through the possible mechanisms mentioned in the previous paragraph.

Finally, our cluster analysis identified BMI subgroups with specific patterns of BMI change and variability, which eventually showed a greater risk of Aβ positivity. Specifically, a subgroup with decreased BMI and a greater variability in BMI was predictive of a higher risk of Aβ positivity. This was again replicated in cluster analyses with various BMI measures and Aβ positivity, a prior finding suggestive of the importance of decreased BMI with greater variability in Aβ positivity.

The strength of this study is that we investigated the associations between Aβ positivity and BMI changes and BMI variability in a large cohort. However, our study has several limitations that need to be addressed. First, due to the inherent challenges of a retrospective cohort study, we did not provide information about the participants’ amyloid status at baseline. Thus, we were not able to show their causal relationships. However, a retrospective cohort study was considered a realistic alternative given that the change in amyloid appears very slowly and the cost of amyloid PET is very expensive. Second, because we used the Asia-Pacific BMI criteria for participants who are obese and underweight, caution should be taken when generalizing our findings to other races. Third, BMI values were collected from March 2020 until 3 years after the Aβ PET scan, and several BMI values were recorded at 3 years after the Aβ PET scan. This limitation might be mitigated to a certain extent with the existing findings that the annual rate of increasing Aβ deposition is very low (Ossenkoppele et al., 2012; Kemppainen et al., 2014; Kim et al., 2016). Fourth, we did not assess the body composition that might explain the associations we reported, such as muscle mass and fat mass. Fifth, we lacked information on whether the BMI change and fluctuations were intentional or unintentional, although these factors may have different effects on Aβ deposition. Sixth, we could not consider the alterations of dietary habit, although these factors might be associated with BMI change and Aβ positivity. Finally, because BMI data were retrospectively derived from the clinical data warehouse, there were differences in the duration of follow-up among participants despite controlling for the duration of follow-up in the process of calculating BMI changes. Instead, the results of the study with data from the clinical data warehouse could reflect the clinical situation in real-world settings, which were considered as real-world evidence for healthcare decisions.

## Conclusion

We provide a comprehensive understanding of the marked influences of BMI change and variability on the risk of Aβ positivity in non-demented individuals. Furthermore, our findings suggest that rigorous strategies for weight maintenance are required to prevent Aβ deposition in non-demented individuals.

## Data availability statement

The raw data supporting the conclusions of this article will be made available by the authors, without undue reservation.

## Ethics statement

The studies involving human participants were reviewed and approved by the Institutional Review Board of the Samsung Medical Center. Written informed consent was obtained from all participants. The patients/participants provided their written informed consent to participate in this study.

## Author contributions

SHK: writing – original draft, review and editing, formal analysis, and data curation. JHK: writing – review and editing, formal analysis, and data curation. YC: conceptualization. BKC, YSC, HJ, HJK, S-BK, and DLN: data curation. KK: methodology, formal analysis, writing – review and editing, and conceptualization. SWS: methodology, writing – review and editing, and conceptualization. All authors contributed to the article and approved the submitted version.

## Conflict of interest

The authors declare that the research was conducted in the absence of any commercial or financial relationships that could be construed as a potential conflict of interest.

## Publisher’s note

All claims expressed in this article are solely those of the authors and do not necessarily represent those of their affiliated organizations, or those of the publisher, the editors and the reviewers. Any product that may be evaluated in this article, or claim that may be made by its manufacturer, is not guaranteed or endorsed by the publisher.

## Footnotes

1
www.R-project.org
