# Supplementary material for: Independent effect of body mass index variation on amyloid-β positivity
Source: Front Aging Neurosci. 2022 Jul 22;14:924550. doi: 10.3389/fnagi.2022.924550 (PMC9354132; doi:10.3389/fnagi.2022.924550)
Supplement: Supplementary file 1 [file Data_Sheet_1.docx]

**Supplementary Material**

**eTable 1.** Measures for BMI variability

**eTable 2.** BMI baseline, change and variability in each subgroup

**eTable 3.** Comparison of related articles, with their BMI definition and outcome

**eFigure 1**. Representative PET images in participants with Aβ (+) and Aβ (-)

**eFigure 2.** Extraction of BMI data in study participants

**eFigure 3.** Three different measures of BMI

**eFigure 4.** Correlation between BMI baseline, change and variability

**eFigure 5.** Plot of silhouette width and AIC and BIC measures

**eTable 1.** Measures for BMI Variability

| Measure | Meaning | Formular |
| --- | --- | --- |
| Mean |  | $\bar{x}=\sum_{i=1}^{n} x_{i}/n$ |
| SD | Standard deviation | $\sqrt{\frac{\sum_{i=1}^{n} (x_{i}-\bar{x})^{2}}{(n-1)}}$ |
| CV | Coefficient of variation | 100 x SD/$\bar{x}$ |
| VIM | Variation independent of mean | 100 x SD/$\bar{x}^{\beta}$ |
| RSD | Residual standard deviation  ($\hat{x}_{1},\hat{x}_{2},...,\hat{x}_{n}$ are the fitted values from a linear regression of BMI against time) | $\sqrt{\frac{\sum_{i=1}^{n} (x_{i}-\hat{x}_{i})^{2}}{(n-2)}}$ |
| ARV | Average real variability | $\frac{1}{n-1}\sum_{i=1}^{n-1} \left\vert x_{i+1}-x_{i} \right\vert$ |
| SV | Successive variation | $\sqrt{\frac{1}{n-1}\sum_{i=1}^{n-1} (x_{i+1}-x_{i}})^{2}$ |

Abbreviation: BMI, body mass index.

**eTable 2.** BMI Baseline, Change, and Variability in Each Subgroup

| Subgroup | BMI | | | | | | | |
| --- | --- | --- | --- | --- | --- | --- | --- | --- |
|  | Baseline | Change | SD | CV | VIM | ARV | SV | RSD |
| 1 | 24.153 | 0.009 | 0.333 | 1.382 | 0.336 | 0.403 | 0.456 | 0.315 |
| 2 | 23.997 | 0.038 | 0.720 | 2.995 | 0.379 | 0.802 | 0.924 | 0.610 |
| 3 | 23.851 | 0.048 | 1.248 | 5.233 | 0.411 | 1.287 | 1.514 | 0.960 |
| 4 | 23.371 | -0.143 | 2.383 | 10.522 | 0.453 | 2.421 | 2.816 | 1.748 |

Abbreviations: ARV, average real variability; BMI, body mass index; CV, coefficient of variation; RSD, residual SD; SV, successive variability; VIM, variability independent of the mean.

Values are presented as mean in each parameter.

**eTable 3.** Comparison of related articles, with their BMI definition and outcome

| Authors (published year), Journal | Subjects | Baseline BMI status | BMI change | BMI variability | Outcome | Main Finding |
| --- | --- | --- | --- | --- | --- | --- |
| Present study | Non-demented | 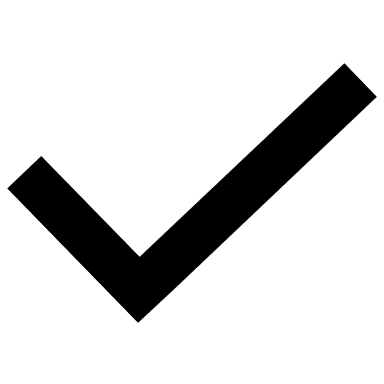 | 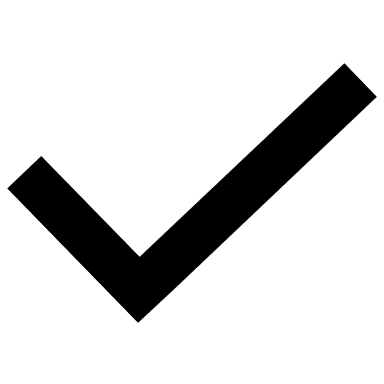 | 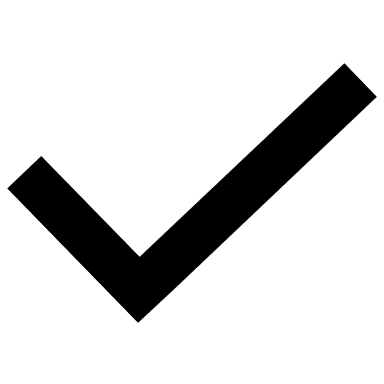 | Presence of Aβ positivity |  |
| (Anstey et al., 2011), Obesity Reviews | Non-demented | 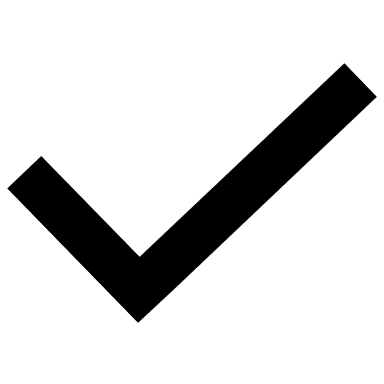 | - | - | Conversion to dementia | Underweight, overweight and obesity in midlife increase dementia risk. |
| (Joo et al., 2018), Front Psychiatry | MCI | 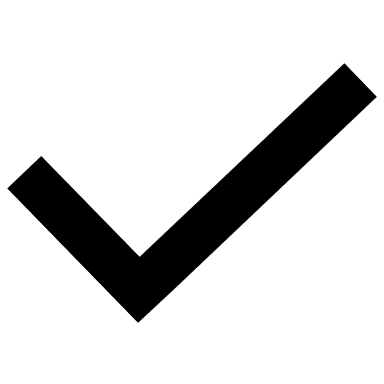 |  |  | Conversion to dementia | Underweight was associated with a higher risk of AD |
| (Ye et al., 2016), JAD | MCI | 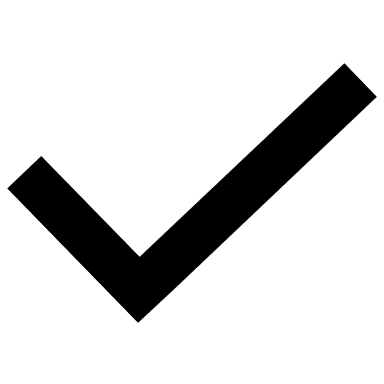 | 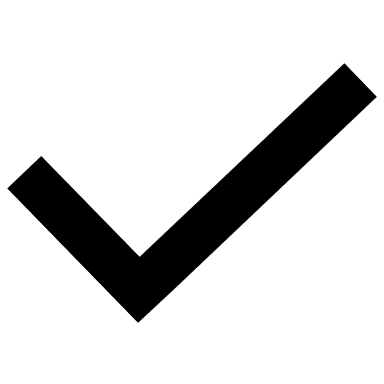 | - | Conversion to dementia | Underweight was associated with a higher risk of AD,  while obesity with lower risk. |
| (Tolppanen et al., 2014), JAD | Non-demented | 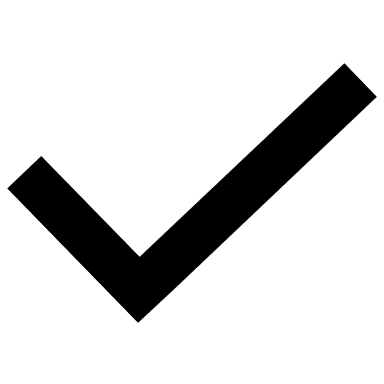 | - | - | Conversion to dementia | Higher midlife BMI is related to higher risk of dementia. |
| (Bell et al., 2017), Nutr Health Aging | Non-demented | 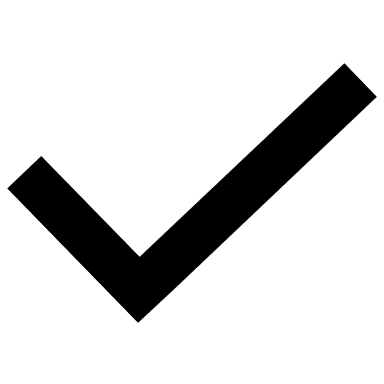 | - | - | Conversion to MCI and dementia | Higher late-life BMI is associated with a lower risk of incident MCI and AD. |
| (Kang et al., 2021), Sci Rep | Non-demented |  | 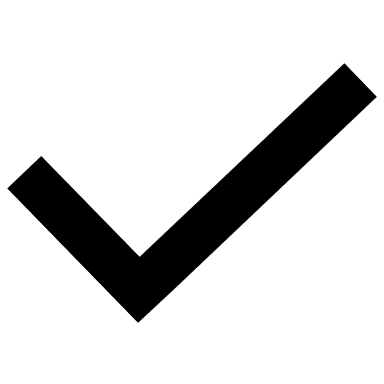 | 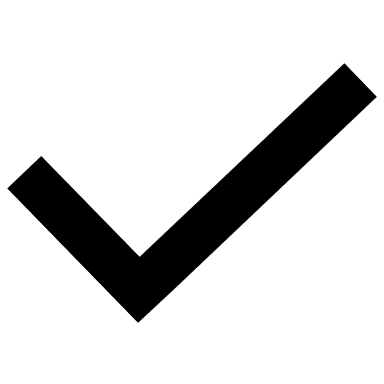 | Conversion to dementia | Decreased BMI is associated with higher risk of AD and higher BMI variability is associated with AD in women |
| (Burns et al., 2010), JAMA neurology | Early AD & Non-demented | 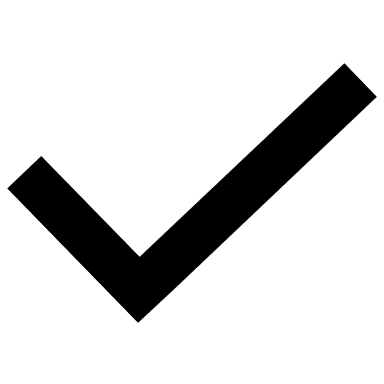 | - | - | Cognitive score and  brain volume | Loss of lean mass is accelerated in AD. |
| (Giudici et al., 2019), Nutrients | Non-demented | - | 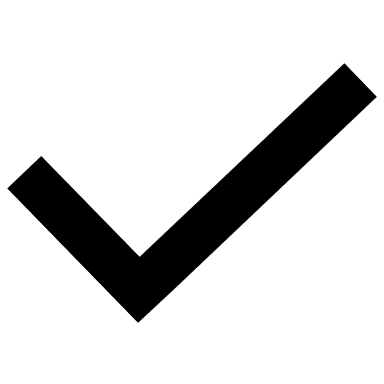 | - | Cognitive score and hippocampal volume | Weight loss ≥5% in the first year predicted higher cognitive decline. |
| (Kim et al., 2015), Int Psychogeriatr | Non-demented | 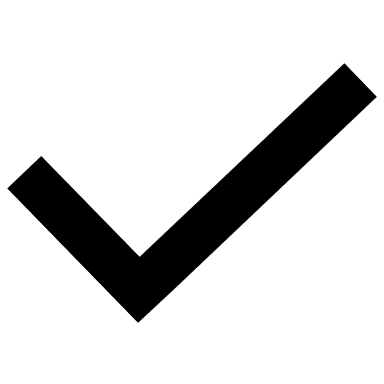 |  |  | Cortical thickness | Lower BMI in late-life is associated with cortical atrophy in men. |
| (Kim et al., 2019), Neurology | Non-demented | 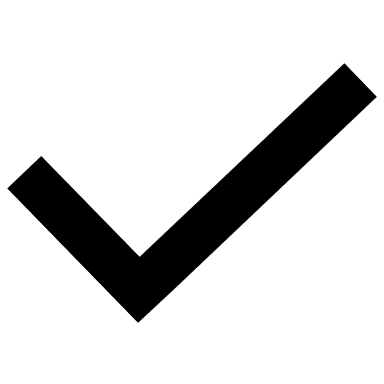 |  |  | Cortical thickness | Lower BMI in late-life is associated with cortical atrophy in men. |
| (Jang et al., 2015), JAD | AD dementia | 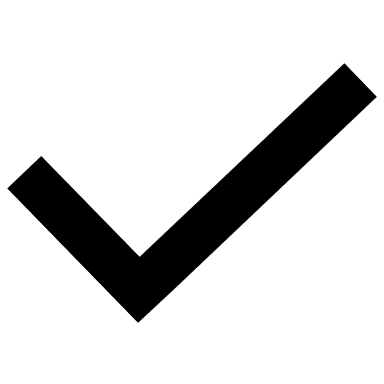 |  |  | mortality | Lower BMI in late-life is associated with higher mortality in patients with AD dementia. |
| (Möllers et al., 2021), ALRT | Non-demented | 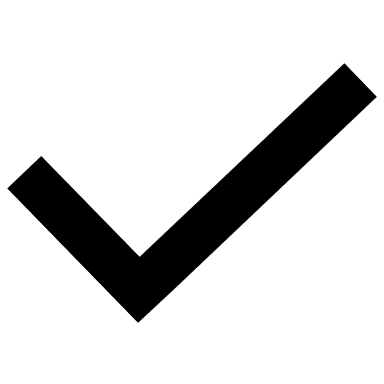 |  |  | Aβ misfolding levels | Lower BMI in midlife is associated with Aβ misfolding. |
| (Buchman et al., 2006), Neurology | Non-demented | 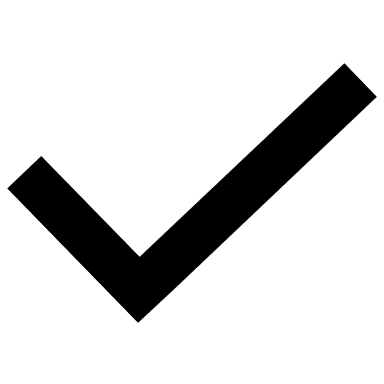 |  |  | Aβ burdens | Lower BMI in late-life is associated with Aβ deposition |
| (Vidoni et al., 2011), Neurology | AD & Non-demented | 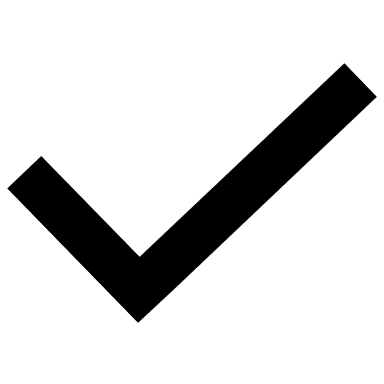 |  |  | Presence of Aβ positivity | Lower BMI in late-life is associated with Aβ positivity in non-demented individuals. |
| (Hsu et al., 2016), JAD | Non-demented | 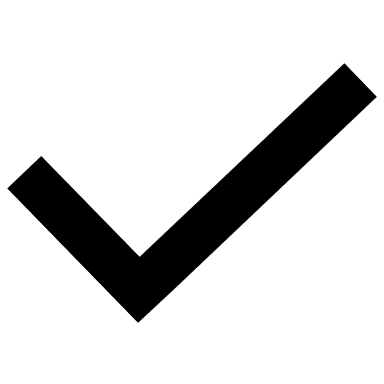 |  |  | Presence of Aβ positivity | Lower BMI in late-life is associated with Aβ positivity |
| (Lee et al., 2020), JAD | Non-demented | 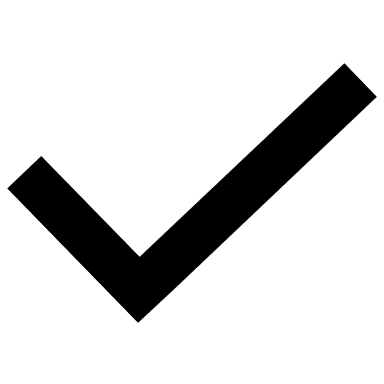 |  |  | Presence of Aβ positivity | Lower BMI in mid-life is associated with Aβ positivity in men |
| (Thirunavu et al., 2019), JAD | Non-demented | 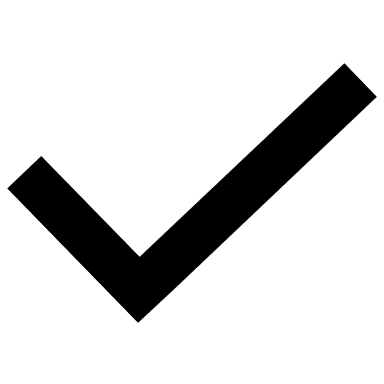 |  |  | Presence of Aβ positivity | Higher BMI in late-life is associated with lower Aβ deposition |
| (Lane et al., 2021), ALRT | Non-demented |  | 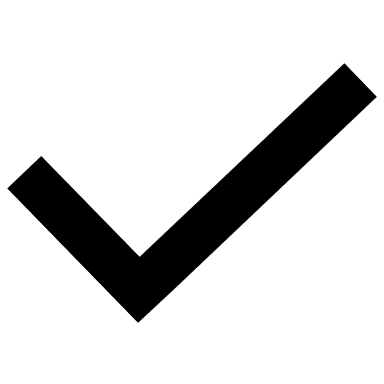 |  | Presence of Aβ positivity | Decreased BMI in late-life is associated with Aβ positivity |
| (Buchman et al., 2021), J Gerontol A Biol Sci Med Sci | Non-demented |  | 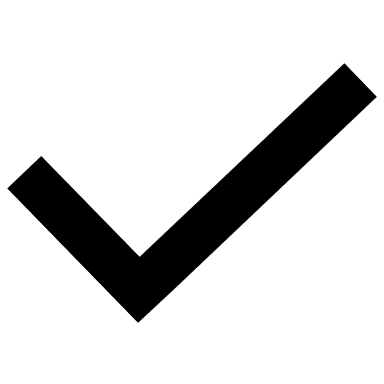 |  | Aβ burdens | BMI change in late-life is associated with amyloid deposition. |

Abbreviations: Aβ, amyloid; AD, Alzheimer’s disease; BMI, body mass index; MCI, mild cognitive impairment.


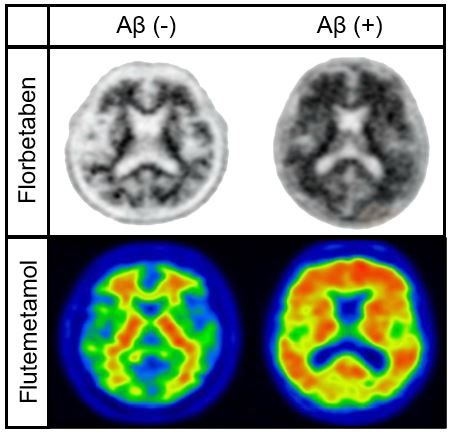


**eFigure 1.** Representative PET images in participants with Aβ (+) and Aβ (-)

Four representative cases of FBB and FMM PET are shown. Aβ (-) images showed no Aβ uptake in the cortex, while Aβ (+) images showed Aβ uptake in the cortex.


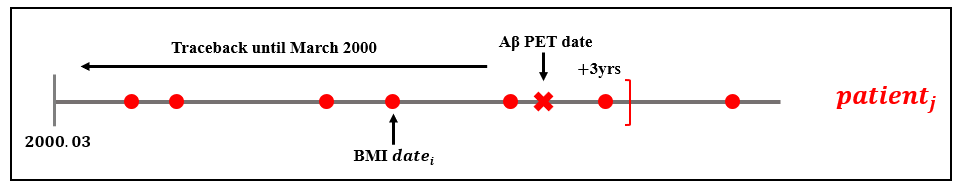


**eFigure 2.** Extraction of BMI Data in Study Participants

For each participant, BMI data were obtained by backtracking their weight and height records in clinical data warehouse of SMC, which were measured at all visits within 3 years after inspecting Aβ PET until March 2000.

Abbreviations: Aβ, amyloid-β; BMI, body mass index.


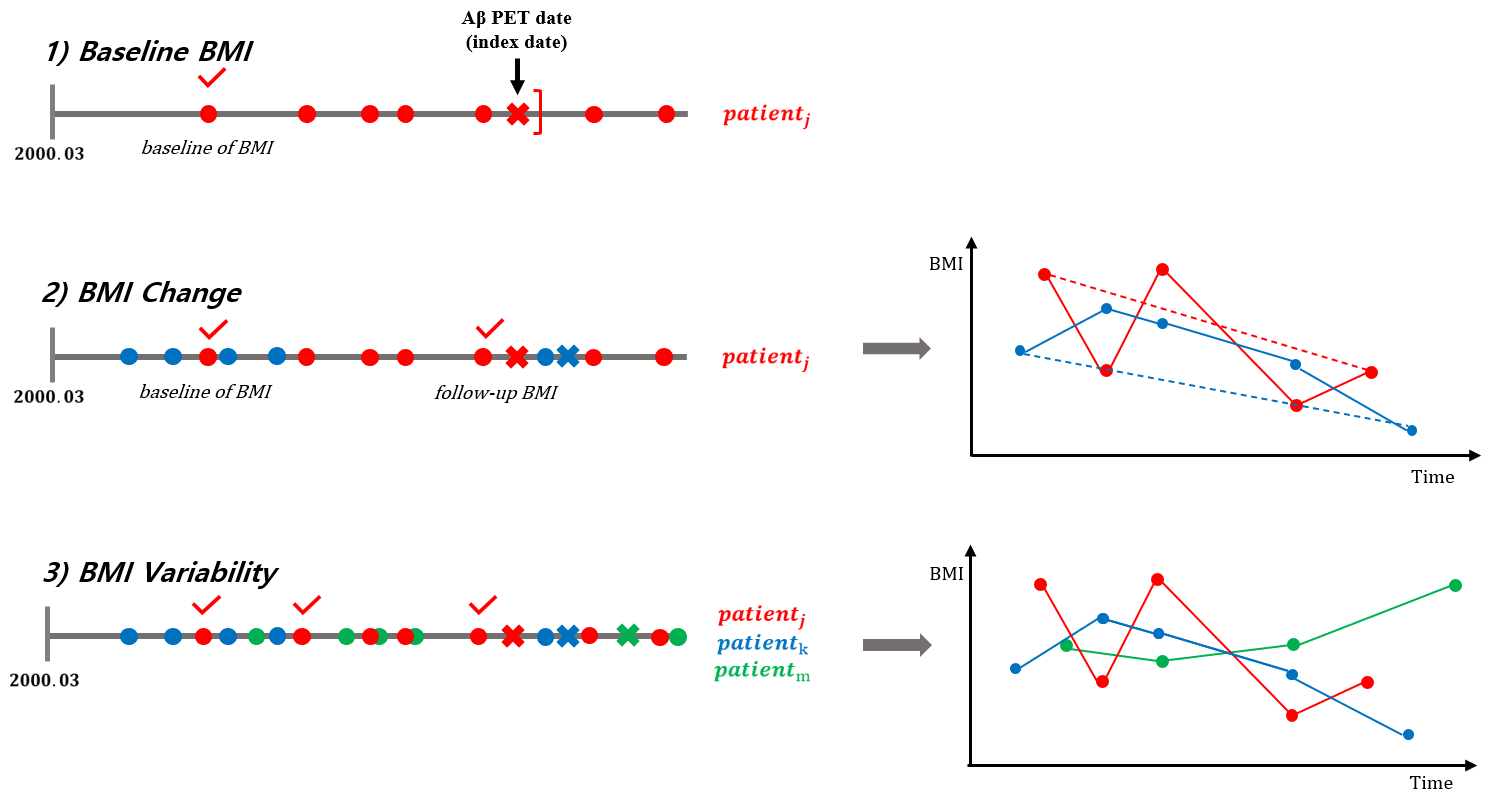


**eFigure 3.** Three Different Measures of BMI

Baseline BMIs was defined as the farthest past measurement from the Aβ PET scan. BMI

change was defined as the change rate by taking the difference between baseline BMI and BMI measured at the closest point to the Aβ PET scan. BMI variability was obtained using BMI values at three or more time points. Abbreviations: Aβ, amyloid-β; BMI, body mass index.


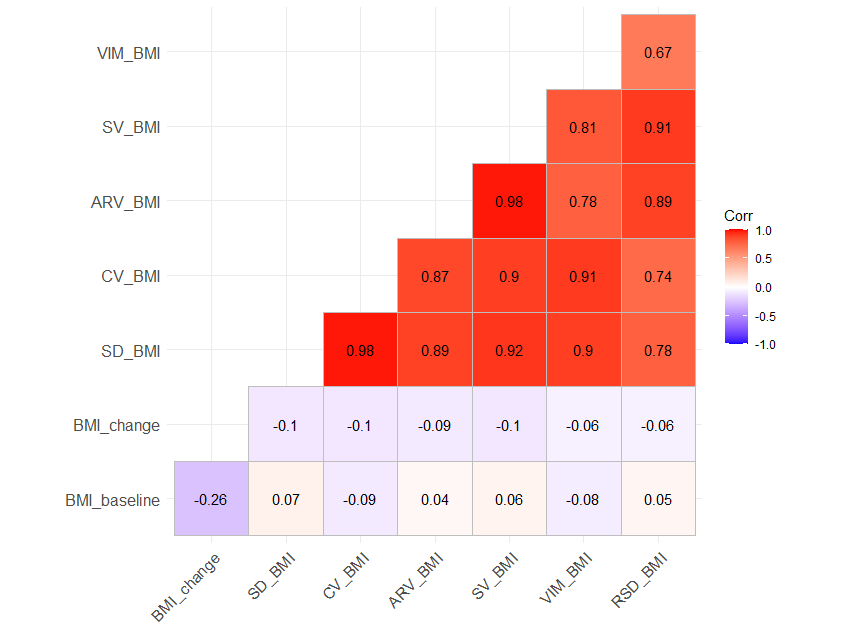


**eFigure 4.** Correlation Between BMI Baseline, Change, and Variability

Correlation coefficient was obtained using Pearson’s correlation analyses among parameters of BMI baseline, change and variability.

Abbreviations: ARV, average real variability; BMI, body mass index; Corr, correlation coefficient; CV, coefficient of variation; RSD, residual SD; SV, successive variability; VIM, variability independent of the mean.


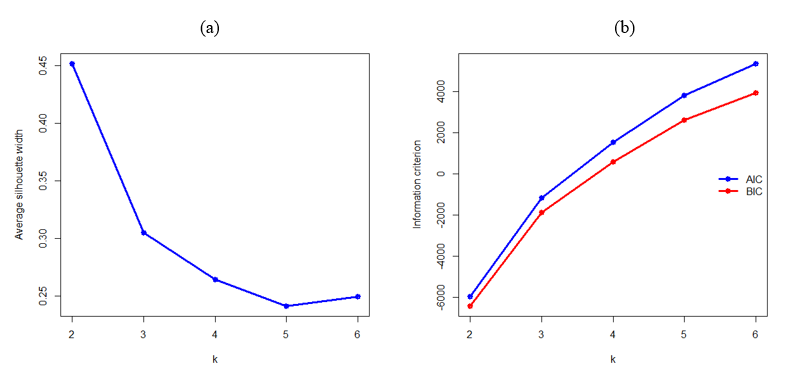


**eFigure 5.** Plot of Silhouette Width and AIC and BIC Measures

The optimal number of clusters was determined by validating clustering results with clinical interpretability as well as the silhouette index, Akaike information criterion (AIC) and Bayesian information criterion (BIC) measures.

(a) Plot of silhouette width in k-means clustering by allowing k to vary from 2 to 6. (b) Plot of the AIC and BIC measures in the GMM by allowing k to vary from 2 to 6.

Abbreviations: AIC, Akaike information criterion; BIC, Bayesian information criterion.

**Reference**

Anstey, K. J., Cherbuin, N., Budge, M., and Young, J. (2011). Body mass index in midlife and late-life as a risk factor for dementia: a meta-analysis of prospective studies. *Obesity reviews : an official journal of the International Association for the Study of Obesity* 12, e426-437. doi: 10.1111/j.1467-789X.2010.00825.x

Bell, S. P., Liu, D., Samuels, L. R., Shah, A. S., Gifford, K. A., Hohman, T. J., et al. (2017). Late-Life Body Mass Index, Rapid Weight Loss, Apolipoprotein E ε4 and the Risk of Cognitive Decline and Incident Dementia. *The journal of nutrition, health & aging* 21, 1259-1267. doi: 10.1007/s12603-017-0906-3

Buchman, A. S., Capuano, A. W., VanderHorst, V., Wilson, R. S., Oveisgharan, S., Schneider, J. A., et al. (2021). Brain β-amyloid links the association of change in BMI with cognitive decline in community-dwelling older adults. *The journals of gerontology. Series A, Biological sciences and medical sciences*. doi: 10.1093/gerona/glab320

Buchman, A. S., Schneider, J. A., Wilson, R. S., Bienias, J. L., and Bennett, D. A. (2006). Body mass index in older persons is associated with Alzheimer disease pathology. *Neurology* 67, 1949-1954. doi: 10.1212/01.wnl.0000247046.90574.0f

Burns, J. M., Johnson, D. K., Watts, A., Swerdlow, R. H., and Brooks, W. M. (2010). Reduced lean mass in early Alzheimer disease and its association with brain atrophy. *Archives of neurology* 67, 428-433. doi: 10.1001/archneurol.2010.38

Giudici, K. V., Guyonnet, S., Rolland, Y., Vellas, B., de Souto Barreto, P., and Nourhashemi, F. (2019). Body Weight Variation Patterns as Predictors of Cognitive Decline over a 5 Year Follow-Up among Community-Dwelling Elderly (MAPT Study). *Nutrients* 11. doi: 10.3390/nu11061371

Hsu, D. C., Mormino, E. C., Schultz, A. P., Amariglio, R. E., Donovan, N. J., Rentz, D. M., et al. (2016). Lower Late-Life Body-Mass Index is Associated with Higher Cortical Amyloid Burden in Clinically Normal Elderly. *Journal of Alzheimer's disease : JAD* 53, 1097-1105. doi: 10.3233/jad-150987

Jang, H., Kim, J. H., Choi, S. H., Lee, Y., Hong, C. H., Jeong, J. H., et al. (2015). Body Mass Index and Mortality Rate in Korean Patients with Alzheimer's Disease. *Journal of Alzheimer's disease : JAD* 46, 399-406. doi: 10.3233/jad-142790

Joo, S. H., Yun, S. H., Kang, D. W., Hahn, C. T., Lim, H. K., and Lee, C. U. (2018). Body Mass Index in Mild Cognitive Impairment According to Age, Sex, Cognitive Intervention, and Hypertension and Risk of Progression to Alzheimer's Disease. *Frontiers in psychiatry* 9, 142. doi: 10.3389/fpsyt.2018.00142

Kang, S. Y., Kim, Y. J., Jang, W., Son, K. Y., Park, H. S., and Kim, Y. S. (2021). Body mass index trajectories and the risk for Alzheimer's disease among older adults. *Scientific reports* 11, 3087. doi: 10.1038/s41598-021-82593-7

Kim, H., Kim, C., Seo, S. W., Na, D. L., Kim, H. J., Kang, M., et al. (2015). Association between body mass index and cortical thickness: among elderly cognitively normal men and women. *International psychogeriatrics* 27, 121-130. doi: 10.1017/s1041610214001744

Kim, S. E., Lee, J. S., Woo, S., Kim, S., Kim, H. J., Park, S., et al. (2019). Sex-specific relationship of cardiometabolic syndrome with lower cortical thickness. *Neurology* 93, e1045-e1057. doi: 10.1212/wnl.0000000000008084

Lane, C. A., Barnes, J., Nicholas, J. M., Baker, J. W., Sudre, C. H., Cash, D. M., et al. (2021). Investigating the relationship between BMI across adulthood and late life brain pathologies. *Alzheimer's research & therapy* 13, 91. doi: 10.1186/s13195-021-00830-7

Lee, S. H., Byun, M. S., Lee, J. H., Yi, D., Sohn, B. K., Lee, J. Y., et al. (2020). Sex-Specific Association of Lifetime Body Mass Index with Alzheimer's Disease Neuroimaging Biomarkers. *Journal of Alzheimer's disease : JAD* 75, 767-777. doi: 10.3233/jad-191216

Möllers, T., Stocker, H., Perna, L., Nabers, A., Rujescu, D., Hartmann, A. M., et al. (2021). Aβ misfolding in blood plasma is inversely associated with body mass index even in middle adulthood. *Alzheimer's research & therapy* 13, 145. doi: 10.1186/s13195-021-00889-2

Thirunavu, V., McCullough, A., Su, Y., Flores, S., Dincer, A., Morris, J. C., et al. (2019). Higher Body Mass Index Is Associated with Lower Cortical Amyloid-β Burden in Cognitively Normal Individuals in Late-Life. *Journal of Alzheimer's disease : JAD* 69, 817-827. doi: 10.3233/jad-190154

Tolppanen, A. M., Ngandu, T., Kåreholt, I., Laatikainen, T., Rusanen, M., Soininen, H., et al. (2014). Midlife and late-life body mass index and late-life dementia: results from a prospective population-based cohort. *Journal of Alzheimer's disease : JAD* 38, 201-209. doi: 10.3233/jad-130698

Vidoni, E. D., Townley, R. A., Honea, R. A., and Burns, J. M. (2011). Alzheimer disease biomarkers are associated with body mass index. *Neurology* 77, 1913-1920. doi: 10.1212/WNL.0b013e318238eec1

Ye, B. S., Jang, E. Y., Kim, S. Y., Kim, E. J., Park, S. A., Lee, Y., et al. (2016). Unstable Body Mass Index and Progression to Probable Alzheimer's Disease Dementia in Patients with Amnestic Mild Cognitive Impairment. *Journal of Alzheimer's disease : JAD* 49, 483-491. doi: 10.3233/jad-150556
